# Soccer heading immediately alters brain function and brain-muscle communication

**DOI:** 10.3389/fnhum.2023.1145700

**Published:** 2023-04-20

**Authors:** Johnny V. V. Parr, Liis Uiga, Ben Marshall, Greg Wood

**Affiliations:** ^1^Department of Sport and Exercise Sciences, Manchester Metropolitan University, Manchester, United Kingdom; ^2^Institute of Sport, Manchester Metropolitan University, Manchester, United Kingdom

**Keywords:** football (soccer), subconcussion, corticomuscular coherence (CMC), mild traumatic brain injury (mTBI), EEG, EMG—electromyogram

## Abstract

**Introduction:**

There is growing evidence of a link between repetitive soccer heading and the increased incidence of neurodegenerative disease. Even a short bout of soccer heading has been shown to impair cognitive performance and disrupt movement control. However, a greater understanding of the mechanisms behind these immediate impairments is needed. The current study attempted to identify how a short bout of soccer heading alters brain function and brain-muscle communication during a movement task.

**Methods:**

Sixty soccer players were exposed to either an acute bout (i.e., 20 balls thrown underarm) of soccer heading (*n* = 30) or a control condition where participants (*n* = 30) headed soccer balls in virtual reality (VR). Before and after heading, we measured cognitive performance on the King-Devick test, as well as electromyography (EMG), electroencephalography (EEG) and brain-muscle communication (i.e., corticomuscular coherence; CMC) during a force precision task.

**Results:**

Following the heading protocol, the VR group improved their cognitive performance whereas the Heading group showed no change. Both groups displayed more precise force contractions at post-test. However, the VR group displayed elevated frontal theta activity and global increases in alpha and beta activity during the contraction task, whereas the Heading group did not. Contrary to our expectations, the Heading group displayed elevated CMC, whereas the VR group showed no change.

**Discussion:**

Our findings indicate a short bout of soccer heading may impair cognitive function and disrupt the organization of efficient neural processes that typically accompany motor skill proficiency. Soccer heading also induced corticomuscular hyperconnectivity, which could represent compensatory brain-muscle communication and an inefficient allocation of increased task-related neuromuscular resources. These initial findings offer insights to the mechanisms behind the impairments experienced after a short bout of repetitive soccer heading.

## 1. Introduction

Recent evidence has suggested that repeated impacts from heading a soccer ball is associated with an increased risk of neurodegenerative disease in later life (Mackay et al., 2019; Ueda et al., 2023). Outfield soccer players are around 3.5 times more likely to suffer from neurodegenerative disease compared to goalkeepers and individuals from the general population matched by age, sex, and socioeconomic status (Russell et al., 2021). This risk rises to a 5-fold increase for those players who played in defensive positions where heading the ball is more frequent (Russell et al., 2021). Exposure to regular soccer heading impacts over a 2 week period can also result in a significant increase in neurological symptoms (Stewart et al., 2017), a finding that becomes concerning given amateur soccer players may be exposed to thousands of heading impacts per year (Lipton et al., 2013).

Despite these alarming statistics, limited research exists to explain the timescale of any neurophysiological changes and whether repeatedly heading a soccer ball induces any immediate changes to brain function (Ntikas et al., 2022). However, there is growing evidence that even a short bout of soccer ball heading can impair cognitive function (Di Virgilio et al., 2016; Ashton et al., 2021). For example, Ashton et al. (2021) examined the effects of 20 soccer headers upon an array of cognitive tasks, such as the King-Devick test, which requires participants to use saccadic eye movements to read a series of numbers as quickly and accurately as possible. Results showed that heading soccer balls resulted in slower performance times and increased errors compared to a control group who did not header soccer balls. Similarly, Nowak et al. (2020) assessed performance on the King-Devick test before and after participants performed either 10 soccer headers or 10 soccer kicks (i.e., no head impacts/control). Whilst both groups performed the test quicker with repeated practice, the magnitude of improvement was significantly attenuated in the heading group who also made more performance errors—an effect that lasted over 24 h. As the King-Devick test has been utilized as a sideline concussion assessment in several contact sports, these findings provide evidence that soccer heading can negatively influence indicators of suspected concussion due to impaired cognitive function.

A short bout of soccer heading can also impair movement regulation. For example, performing 10 soccer headers has been shown to disrupt indices of postural control for up to 24 h (Haran et al., 2013; Hwang et al., 2017), suggesting a disturbance to vestibular processing and the ability to upregulate visual feedback to guide motor actions (Hwang et al., 2017). Interestingly, Hwang et al.’s (2017) findings indicate that disturbances to postural control caused by heading were expressed as an absence of a learning effect on their balance task that was observed in their control group. The tendency for soccer heading to attenuate learning effects in cognitive tasks (i.e., King-Devick) may therefore extend to motor control tasks. There is also evidence that the detrimental effects of heading might be underpinned by fundamental changes to neurophysiological activity. For example, performing a bout of 20 repetitive soccer headers has been shown to alter motor-evoked potentials (MEP) triggered by transcranial magnetic stimulation (TMS) over the primary motor cortex (Di Virgilio et al., 2016), suggesting altered corticomotor inhibition and impaired efficiency of brain-muscle interactions. Whilst 20 repetitive headers is above the average number of headers reported per 90 min in elite level football (e.g., central defenders = 7.5) (The Football Association, 2021a), it is aligned with the upper range of headers previously recorded from training sessions with adolescent players (Koerte et al., 2017). This suggests that soccer players might regularly be experiencing similar neurophysiological responses to heading.

Despite these findings, the link between impaired movement and altered neurophysiological activity following a short bout of heading remains underexplored. If soccer heading causes altered neurophysiological activity during movement, we might expect associated changes to specific corticomuscular features that are associated with increased movement proficiency. For example, electroencephalographic (EEG) theta activity (∼5–7 Hz) across the frontal region of the cortex is associated with cognitive control (Cavanagh and Frank, 2014) and has been shown to be heightened in expert versus novice golfers (Baumeister et al., 2008), during the “flow-state” of mental tasks (Katahira et al., 2018), and when optimally controlling attention during an isometric force precision task (Parr et al., 2023). EEG activity across the alpha (∼8–12 Hz) frequency band also plays an active role in motor control by exerting regional inhibition across the cortex, with increased alpha activity reflecting increased cortical inhibition (Jensen and Mazaheri, 2010). Increased movement proficiency has been associated with elevated alpha activity in a manner consistent with neural efficiency, whereby effective task performance is achieved with a general reduction in cognitive effort that manifests as a quiescence of cortical activation (Del Percio et al., 2009; Babiloni et al., 2010; Parr et al., 2021). Finally, the realization of precise steady-state motor output has been associated with increased EEG beta activity (∼15–30 Hz) and elevated beta-range corticomuscular coherence (CMC) between electromyographic (EMG) activity of the contracting limb and EEG activity across the contralateral sensorimotor cortex (Kristeva et al., 2007). Whilst EEG beta activity is linked to sensorimotor inhibition and “signalling the status quo” (Engel and Fries, 2010), beta CMC is proposed to reflect the cortical control of motor unit firing via the direct corticospinal pathway, and the transmission of beta oscillations from the cortex to periphery to maintain a stable sensorimotor output (Mima and Hallett, 1999). Taken together, these corticomuscular features have each been associated with movement proficiency and should therefore accompany practice-related improvements on a motor task. Given that a short bout of soccer heading has been shown to impair motor control, attenuate motor learning (Haran et al., 2013; Hwang et al., 2017), and alter cortical and corticospinal activity (Di Virgilio et al., 2016), we could expect a similar soccer heading protocol to disturb or attenuate the corticomuscular responses that would be typically observed in response to practice.

The aim of this study was to explore how a short bout of repetitive soccer heading alters the cortical and corticomuscular control of movement. To achieve this, we measured EEG and EMG activity during an isometric force precision task, before and after participants performed 20 soccer headers of either a real (experimental group) or a virtual (control group) soccer ball. We also measured cognitive function using the King-Devick test. In line with previous research, we expected our control group to display practice-induced improvements on the King-Devick test, as expressed by faster completion times and fewer errors. We also expected the control group to display more accurate and precise isometric force contractions from pre- to post-test, accompanied by increased frontal theta activity, increased alpha and beta activity, and increased beta CMC. By contrast, we expected these practice-induced adaptions to be absent or attenuated in the group who were exposed to soccer heading.

## 2. Materials and methods

### 2.1. Participants

Sixty soccer players were recruited to take part in the study and were assigned to either a control “virtual reality (VR)” group (15 males and 15 females; Mean age = 25.73 years, *SD* = 6.11 years) or an experimental “Heading” group (15 males and 15 females; Mean age = 23.53 years, *SD* = 5.25 years) in a pseudo-randomized fashion. The playing level of participants included recreational (*n* = 47), county (*n* = 7), and semi-professional (*n* = 6). The playing positions of participants included goalkeepers (*n* = 5), defenders (*n* = 24), midfielders (*n* = 26) and forwards (*n* = 5). All participants reported no prior history of concussion. A full demographic breakdown of participants can be found in our supplementary material available on the Open Science Framework.^1^ Whilst it is currently unclear how soccer heading immediately alters EEG and CMC activity, previous research has reported large effects (η*p*^2^ = 0.43) of soccer heading upon cognitive function (Ashton et al., 2021). If assuming a large effect with 80% power and an alpha of *p* = 0.05 a total sample size of 14 would have been required to run a mixed-model 2 × 2 repeated measures ANOVA (calculated G*POWER software 3.1; Henrich University Dusseldorf, Germany). However, we chose to increase our sample size to enable the detection of more subtle effects given the novelty of our study. *Post-hoc* sensitivity analyses therefore indicated that with 60 participants and an alpha of *p* = 0.05, 80% power is achieved for small to medium effects size (η*p*^2^ > 0.032). All participants were asked to refrain from playing soccer for 24 h prior to testing and to refrain from any activities that involve head impacts.

### 2.2. Experimental procedure

Participants attended the laboratory for approximately 2 h. After providing written informed consent participants completed the King-Devick test (Galetta et al., 2011) and were asked to indicate the extent to which they were currently experiencing a list of concussive symptoms. Participants were then fitted with EEG and EMG equipment before performing two maximum voluntary contractions (MVC’s) followed by the neurophysiological assessment (described below). The control (VR) group then headed a virtual soccer ball 20 times in VR using the Rezzil Player 22 (Rezzil Europe, Manchester, UK) application on the Oculus Quest 2 head-mounted display (HMD; Facebook Technologies, Menlo Park, CA, USA). This hardware displays at a resolution of 1,832 × 1,920 pixels per eye at 120 Hz and has a field of view of 89°. The ball was projected to the players from a virtual ball machine, and they were required to perform a defensive header, heading the ball back as far past the virtual ball machine as possible whilst keeping both feet on the floor (i.e., jumping was not permitted). Following the protocol of previous studies (Ashton et al., 2021) the experimental (Heading) group headed a standard (size five) Mitre Impel soccer ball 20 times that was inflated to the maximum allowed by the IFAB (16.2 psi). In this condition, the ball was thrown underarm to each participant from 13 m with participants required to also perform a defensive header, heading as far past the thrower as possible whilst keeping both feet on the floor. *Post-hoc* video analysis of 40 underarm throws using the Tracker Video Analysis and Modeling Tool (Brown, 2009) indicated that the typical ball velocity was ∼8.10 m/s with a standard deviation of 0.48 m/s. An accelerometer was attached to a strap at the back of the head and aligned with inion near the base of the skull in all conditions. After completing their respective heading conditions, participants again completed the King-Devick assessment, self-reported their concussive symptoms, and were refitted for EEG and EMG analyses for the neurophysiological assessment. The heading activities lasted approximately 5 min in total for each group and the time between the heading conditions and post-test neurophysiological assessments was standardized to 30 min across groups.

### 2.3. Materials and measures

#### 2.3.1. Self-report

At pre and post-test, participants were asked two questions, (1) “On a scale of 0–10, how much confusion, discomfort, drowsiness, ringing in your ears or pain do you feel in your head at this current moment?” and (2) “Which one symptom best describes how you feel currently? Confusion, Discomfort, Drowsiness, Ringing in ears, Pain, N/A (no symptoms), Other”.

#### 2.3.2. King-Devick test

The King-Devick test measures saccadic eye speed and provides an immediate, low-cost indicator of head trauma or suspected concussion (Galetta et al., 2011). The test consists of a series of horizontally spaced numbers at variable distances within columns and rows displayed on A4 sized cards. Participants had to read aloud the numbers running from top-left to bottom-right as quickly as possible. A stopwatch was used by the experimenter to measure the start and end times and the number of errors for each test card. Participants first practiced with a demonstration card before they completed three test cards that increased in difficulty. The time taken to complete each test card was accumulated to give a total test time for each participant at pre and post-test.

#### 2.3.3. Head acceleration

Prior to heading, participants wore a GENEActiv triaxial accelerometer that was attached to a strap at the back of the head and aligned with inion near the base of the skull. Acceleration was expressed in *g*’s (retaining the earth’s gravitational pull) with signals sampled at 100 Hz and smoothed using a 4th-order, Butterworth filter (dual-pass) with a low-pass cut-off frequency of 50 Hz (Wu et al., 2016). The traces of the resultant linear accelerations from the heading activity were visually inspected for definitive peaks that equated to either the acceleration of the head (VR group) or impact with the ball (Heading group), which determined the mean peak linear acceleration.

#### 2.3.4. Neurophysiological assessment

Using an experimental task we have implemented previously (Parr et al., 2022, 2023), participants were instructed to squeeze a dynamometer by contracting their right hand at 15 ± 5% of their MVC for 5 s. The force produced and target force boundaries (i.e., ±5%) were displayed in real time on a monitor using Labchart 8.0. Following a familiarization block of 10 trials, participants completed 50 contractions, divided into five blocks of 10 contractions. Each 5 s contraction was followed by a 5 s rest, and each block was separated by a 30 s rest period to minimize muscular fatigue. The onset and offset of each 5 s contraction were indicated by auditory tones (10 ms duration) controlled by PsychoPy (Peirce, 2007) and used as event triggers for the physiological and kinetic data.

#### 2.3.5. Electrophysiological and kinetic data

The EEG signals were recorded from 29 active shielded AgCl electrodes embedded in a stretchable fabric cap (eego sports, Ant Neuro, Hengelo, Netherlands) and positioned according to the extended 10–20 international system (Jurcak et al., 2007). Electrodes in sites CPz and AFz were used as reference and ground, respectively. Naison, Inion, and preauricular points were used as anatomical landmarks to position the EEG cap. Conductive gel for electrophysiological measurements was used (Signa gel, Parker), and impedance was kept below 20 kΩ. The EMG signals were recorded from a pair of bipolar surface EMG electrodes (eego sports, Ant Neuro, Hengelo, Netherlands) placed on the skin to record activity from extensor carpi radialis of the right forearm according to the guidelines set out by SENIAM.^2^ The participant’s right forearm was then strapped to a cushioned rig positioned on the desk, where participants held a dynamometer connected to a PowerLab 4/25T (AD Instruments, Bella Vista, NSW, Australia) to record hand contraction force (in kilograms) via Labchart 8 software (ADinstruments). The force, EEG, and EMG signals were recorded at a sample rate of 1,000 Hz and were synchronized through a square-wave trigger (i.e., time point zero in subsequent analyses) sent by a LabJack U3 device (LabJack Corporation, Lakewood, CO, United States) at each auditory contraction prompt generated through a bespoke Psychopy programme.

#### 2.3.6. Data processing

For EMG-specific analyses, signals were down-sampled (500 Hz), band-pass filtered (10–200 Hz, FIR [finite infinite response]), and notch filtered (48–52 Hz) prior to being cut into epochs ranging from −2 to +6 s relative to the onset auditory stimulus. For EEG and CMC analyses, signals were down sampled (250 Hz) and band-pass filtered (1–45 Hz; finite impulse response) prior to being cut into epochs ranging from −2 to +6 s relative to the onset auditory stimulus. Epochs were then baseline corrected by subtracting the mean activity occurring −1,500 ms to −500 ms prior to the auditory stimulus. These epochs were visually inspected, and those showing large EEG contamination from muscular artifacts were discarded (from both EEG and EMG analyses). No bad EEG channels were identified. Independent component analysis (ICA) weights were obtained through the RunICA infomax algorithm (Jung et al., 1998) running on EEG signals. ICA weights that presented obvious non-neural activity upon visual inspection (e.g., eye blinks, line noise, and muscular artifact) were manually rejected. The EMG data for one participant was found to be strongly contaminated by line noise, resulting in their data for EMG activity and CMC analyses being excluded from further analyses. These processing steps were performed using EEGLAB functions (Delorme and Makeig, 2004) for MATLAB. Given that beta CMC is known to occur during steady-state (but not dynamic) phases of force contraction (Omlor et al., 2007), we focused our analysis of force, EEG and EMG data across the window occurring between 1 and 5 s following the onset stimulus. The initial 1,000 ms were not considered to minimize data containing initial dynamic contractions.

#### 2.3.7. Force control

Force control was assessed as the steadiness of each contraction during the steady-state phase of the task (i.e., between 1 and 5 s post-stimulus). Steadiness was defined as the coefficient of variance (CoV), calculated as the ratio between standard deviation and mean during the steady phase. The post-test data for one participant was corrupted offline resulting in their data (pre- and post-test) being subsequently removed from further analyses.

#### 2.3.8. EEG power

Time-frequency decomposition was performed through a short-time Fast Fourier Transform (FFT) conducted on 129 overlapping windows, each of 0.5 s length, with central point’s ranging from −1.75–5.75 s relative to the onset of the auditory go stimulus. Prior to FFT, data points in each window were Hanning tapered and zero padded to reach 1 s. This procedure generated complex-valued coefficients in the time-frequency plane with a precision of 58.6 ms and 1 Hz. EEG power (μV2) was calculated for the theta (5–7 Hz), alpha (8–12 Hz) and beta (15–30 Hz) frequency bands as the squared amplitude of each FFT coefficient, which was then averaged across the 69 overlapping segments spanning the steady phase.

#### 2.3.9. EMG activity

Muscular activity was calculated as the root-mean-square (RMS) of the EMG signal occurring across the steady phase (i.e., 1–5 s post-stimulus) for each trial. Data were normalized for each participant by dividing trial-level RMS by the single largest RMS trial value recorded across all experimental trials (i.e., % of max RMS).

#### 2.3.10. Corticomuscular coherence (CMC)

Coherence between the EEG and EMG signals were calculated by magnitude-squared coherence across the steady-state task phase (Mima and Hallett, 1999; Parr et al., 2023). The coherence function provides a normative measure of linear correlation on a scale from 0 to 1, where 1 indicates a perfect linear correlation. For multiple corrections across the 16 frequency bins (i.e., between 15 and 30), we applied the standard Bonferroni correction to define the significance level of coherence (Rosenberg et al., 1989). Thus, with 16 frequency bins, 69 overlapping segments, and an alpha of 95%, the significance level for our analyses was determined to be 0.0813. We measured the area under the coherence curve and above the significance level in the beta frequency range. This procedure addresses the overall effect within frequency bands on interest (Chakarov et al., 2009) and considers potential shifts in the location of peak coherence across trials, meaning that essential information on the correlated activity between EEG and EMG activity does not get overlooked (Johnson and Shinohara, 2012).

### 2.4. Statistical analysis

Our primary EEG and CMC analysis was focused on channel C3 as it overlies the hand area of the primary sensorimotor cortex responsible for right hand contractions. The Gaussian distribution of data was checked via Shapiro–Wilk. For parametric data (King-Devick times, force control, EMG activity, and C3 CMC), a series of 2 (Group; Heading vs. VR) × 2 (Time; pre vs. post) repeated measures analyses of variance (ANOVA) were conducted with Bonferroni corrected pairwise comparisons used to probe significant effects *post-hoc*. To achieve a normal distribution, data for King-Devick time and force control were log-transformed. Where a normal distribution of data could not be achieved (EEG spectral analyses, head impact force, and King-Devick errors), Wilcoxon signed-ranks tests were used to determine significant changes from pre-test to post-test for each group separately, and Mann–Whitney U tests were used to determine significant differences between groups at both pre- and post-test. Effect sizes for non-parametric tests were reported as *r*, with values of 0.1, 0.3, and 0.5 reflecting small, medium, and large effects, respectively (Cohen, 1992). Whilst we focused our primary EEG analyses on specific channels (e.g., Fz for frontal theta and C3 for alpha, beta, and CMC) we also performed exploratory analyses based on topographical inspection of statistical difference maps between pre- and post-test. Where exploratory analyses required many channel-wise comparisons (i.e., one test for many channels), acquired *p*-values were adjusted using the Benjamini–Hochberg correction of the false discovery rate (FDR).

## 3. Results

### 3.1. Self-report

In the Heading group, 27/30 participants (90%) experienced symptoms related to concussion whereas in the VR group only 3/27 participants (10%) reported similar symptoms (Table 1).

**TABLE 1:** Self-report data on the severity and description of concussion symptoms pre and post-test for each experimental condition.

Group	Concussion severity score (/10)		Symptom description
	Pre	Post	
VR	Median = 0.00	Median = 0.00	Headache (*n* = 1)
	Mean = 0.13	Mean = 0.13	Head pressure (*n* = 1)
	Min = 0.00	Min = 0.00	Drowsy (*n* = 1)
	Max = 2.00	Max = 1.00	None (*n* = 27)

Heading	Median = 0.00	Median = 3.00	Discomfort (n = 14)
	Mean = 0.20	Mean = 2.97	Pain (*n* = 5)
	Min = 0.00	Min = 0.00	Drowsy (*n* = 3)
	Max = 2.00	Max = 7.00	Ringing in ears (*n* = 2)
			Confusion (*n* = 1)
			Blood pumping (*n* = 1)
			Head pressure (*n* = 1)
			None (*n* = 3)

### 3.2. Head acceleration

**FIGURE 1:**
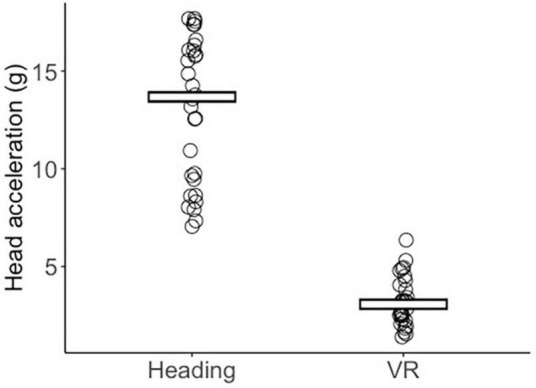
Jitter plot displaying each participant’s mean peak head acceleration (*g*) across the Heading and VR conditions. Crossbars reflect the group median.

A Mann–Whitney U test indicated that head accelerations were significantly greater (*Z* = 6.653, *p* < 0.001, *r* = 1.215) in the Heading group (median = 13.68 *g*, IQR = 3.75) compared to the VR group (median = 3.07 *g*, IQR = 1.66; Figure 1).

### 3.3. King-Devick

**FIGURE 2:**
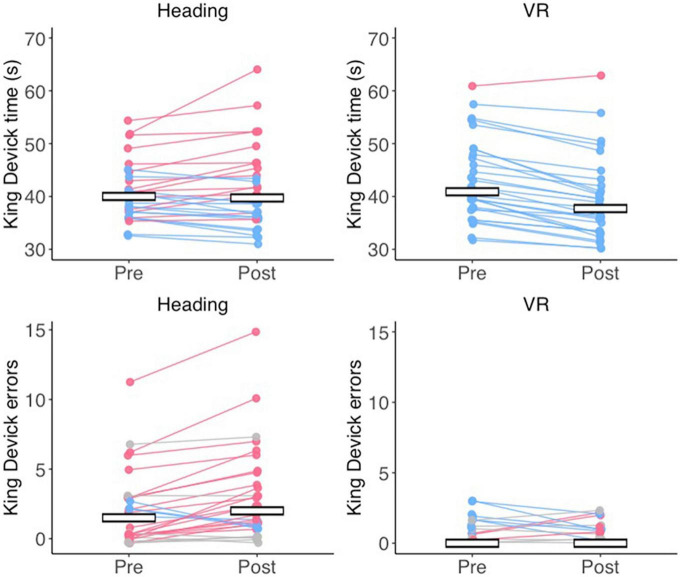
Cognitive impairments after an acute bout of soccer heading. Jitter plots displaying the mean time (in seconds) taken to complete the King Devick **(top row)** and the number of errors **(bottom row)** at pre- and post-test for the Heading and VR groups. Lines connect the pre- and post-test times for a single participant, with red data reflecting slower times/more errors at post-test, blue data reflecting faster times/less errors at post-test, and gray data refleting no change. Crossbars indicate the group mean for King-Devick time, and the group median for King-Devick errors.

A significant main effect of time was found for King-Devick completion times [*F*(1, 58) = 27.884, *p* < 0.001, η*p*^2^ = 0.325] that was superseded by a significant group x time interaction (*F*(1, 58) = 39.807, *p* < 0.001, η*p*^2^ = 0.407). Bonferroni adjusted *post-hoc* comparisons revealed that the VR group performed significantly faster at post-test compared to pre-test (*p* < 0.001) whereas the Heading group displayed no change (*p* < 0.470; Figure 2). The main effect of group was non-significant [*F*(1, 58) = 0.029, *p* = 0.866, η*p*^2^ = 0.000]. For King-Devick errors, Wilcoxon signed-ranks tests indicated that the Heading group displayed significantly more errors at post-test (median = 2.00, IQR = 3.75) compared to pre-test (median = 1.50, IQR = 3.00; *Z* = 3.194, *p* = 0.001, *r* = 0.583) whereas the VR group displayed no change (*Z* = 1.498, *p* = 0.134, *r* = 0.274) from pre (median = 0.00, IQR = 1.00) to post-test (median = 0.00, IQR = 1.00). Mann–Whitney U tests further indicated no difference in errors between groups at pre-test (*Z* = 1.652, *p* = 0.099, *r* = 0.302), but significantly more errors in the Heading group compared to the VR group at post-test (*Z* = 4.117, *p* < 0.001, *r* = 0.752; Figure 2).

### 3.4. Force control

A significant main effect of time was observed for force control, *F*(1, 57) = 14.875, *p* < 0.001, η*p*^2^ = 0.207, with participants displaying steadier contractions at post-test (6.59 ± 3.69 %) compared to pre-test (7.51 ± 3.65%). There was no main effect for group [*F*(1, 57) = 0.381, *p* = 0.539, η*p*^2^ = 0.007] and no time x group interaction [*F*(1, 57) = 0.144, *p* = 0.705, η*p*^2^ = 0.003].

### 3.5. EMG activity

ANOVA revealed a significant main effect of time, *F*(1, 57) = 4.886, *p* = 0.031, η*p*^2^ = 0.079, with activity of the forearm extensor muscle decreasing from pre- (77.22 ± 8.17%) to post-test (74.22 ± 10.84%). There was no main effect of Group [*F*(1, 57) = 0.908, *p* = 0.345, η*p*^2^ = 0.016] and no Group x Time interaction [*F*(1,57) = 0.000, *p* = 0.994, η*p*^2^ = 0.000].

### 3.6. Frontal theta power

For channel Fz, a Wilcoxon signed-ranks test indicated that the VR group displayed significantly increased theta activity from pre- to post-test (*Z* = 2.27, *p* = 0.023, *r* = 0.414), whereas the Heading group displayed no change (*Z* = −0.689, *p* = 0.491, *r* = 0.126). Mann–Whitney U tests confirmed no differences in Fz theta activity between the VR and Heading groups at pre-test (*p* = 0.569) and post-test (*p* = 1.00). Topographical inspection of data suggested the increase in frontal theta activity in the VR group occurred across a cluster of channels spanning the frontal region. Exploratory analyses confirmed that the VR group also displayed significantly increased theta activity from pre- to post-test across channels F4 (*Z* = 2.458, *p* = 0.014, *r* = 0.449) and F3 (*Z* = 3.137, *p* = 0.002, *r* = 0.573).

### 3.7. Alpha power

For channel C3, a Wilcoxon signed-ranks test indicated that the VR group displayed significantly increased alpha activity from pre- to post-test (*Z* = 3.157, *p* = 0.002, *r* = 0.576), whereas the Heading group displayed no change (*Z* = 0.504, *p* = 0.614, *r* = 0.092). No differences in C3 alpha activity were observed between groups at pre-test (*p* = 0.223) or post-test (*p* = 0.176). Exploratory analyses comparing channel-wise differences in alpha activity from pre- to post-test indicated that the VR group displayed a global increase in alpha activity spanning 23 of 29 channels (*ps* ≤ 0.015).

### 3.8. Beta power

For channel C3, a Wilcoxon signed-ranks test indicated that the VR group displayed significantly increased beta activity from pre- to post-test (*Z* = 2.869, *p* = 0.004, *r* = 0.524), whereas the Heading group displayed no change (*Z* = −0.751, *p* = 0.453, *r* = 0.083). No differences in C3 beta activity were observed between groups at pre-test (*p* = 0.819) or post-test (*p* = 0.620). Exploratory analyses comparing channel-wise differences in beta activity from pre- to post-test indicated that the VR group displayed a global increase in beta activity spanning 16 of 29 channels (*ps* < 0.007).

### 3.9. Corticomuscular coherence

**FIGURE 3:**
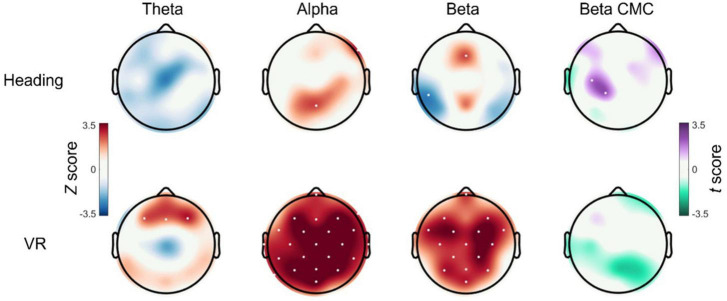
Cortical and corticomuscular changes after an acute bout of heading. Topoplots displaying the EEG and CMC statistical difference maps between pre- and post-test for both the Heading **(top row)** and VR groups **(bottom row)**. For theta, alpha, and beta activity, non-parametric Wilcoxon signed-ranks tests were used to generate channel-wise Z scores, with red cortical areas indicating an increase in activity from pre- to post-test and blue cortical areas indicating a reduction in activity from pre- to post-test. For beta CMC, paired samples *t*-tests were used to generate channel-wise t-scores, with purple cortical areas indicating increased CMC from pre- to post-test and green cortical areas indicating decreased CMC from pre- to post-test. Channels that displayed a significant change from pre- to post-test are indicated with white dots.

A significant group × time interaction was observed for C3 CMC, *F*(1, 57) = 5.631, *p* = 0.021, η*p*^2^ = 0.090. Bonferroni corrected pairwise comparisons indicated that the Heading group displayed significantly increased C3 CMC from pre-test (*M* = 0.52 ± 0.06) to post-test (*M* = 0.56 ± 0.06, *p* = 0.026) whereas the VR group showed no change (*p* = 0.293). There were no significant main effects of Group [*F*(1, 57) = 0.361, *p* = 0.550, η*p*^2^ = 0.006] or Time [*F*(1, 57) = 0.780, *p* = 0.381, η*p*^2^ = 0.013]. Topographical inspection of data confirmed that the increased CMC observed in the Heading group was localized to the left motor cortex, with the effect appearing to extend across channel CP1. Exploratory analyses confirmed a group x time interaction for channel CP1, *F*(1, 57) = 6.050, *p* = 0.017, η*p*^2^ = 0.096, with the Heading group again displaying significantly increased CP1 CMC from pre-test (*M* = 0.506 ± 0.05) to post-test (*M* = 0.541 ± 0.05, *p* = 0.011) and the VR group displaying no change (*p* = 0.408). Topoplots for these data can be seen in Figure 3.

## 4. Discussion

The aim of this study was to examine the effects of an acute bout of soccer heading on brain function and brain-muscle communication during a movement task. As expected, the impact of heading a soccer ball exposed players to significantly higher (75% higher) linear accelerations of the head compared to heading a ball in VR. The Heading group also reported an array of symptoms commonly associated with concussion. While the VR group completed the King-Devick significantly faster post-test, the Heading group showed no improvements and made more errors. These impairments in the ability to improve on the King-Devick test have been reported in previous research and have been suggested to highlight an impairment in cognitive function that reduces the ability to learn such tasks (Nowak et al., 2020).

Interestingly, both the Heading and VR groups displayed comparable improvements in movement control from pre- to post-test. This finding is contrary to previous research indicating that repetitive heading can impair motor performance (Haran et al., 2013; Hwang et al., 2017) or the ability to improve motor performance in response to practice (Hwang et al., 2017). However, our EEG data suggest that these improvements were underpinned by fundamentally different neurophysiological activity. For example, the VR group displayed elevated frontal theta activity and a global increase alpha activity. These collaborative neural responses may individually reflect enhanced cognitive control [frontal theta; (Cavanagh and Frank, 2014)], and reduced cortical activation [alpha; (Del Percio et al., 2009; Parr et al., 2021; Fang et al., 2022)], that collectively indicate a “relaxed and focused brain” and the efficient neural control of movement in response to practice (Filho et al., 2021). The VR group also displayed increase beta activity which, in the context of isometric tasks, promotes inhibitory motor control to support force precision and steadiness (Kristeva et al., 2007; Engel and Fries, 2010). The absence of these spectral EEG changes in the Heading group may indicate that an acute bout of soccer heading interfered with these efficiency-related neurophysiological adaptations. Interestingly, a recent systematic review found the negative effects of soccer heading upon cognitive performance and motor control to occur inconsistently (Snowden et al., 2021). Our findings could, therefore, indicate that individuals might adopt latent compensatory strategies or heightened cognitive effort to overcome concussive effects, a response to soccer heading impact that might not be captured by cognitive or behavioral assessments alone. However, there remains a paucity of evidence exploring how soccer heading immediately alters cortical activity, so our findings should be taken with caution until future work can display a replication of these findings.

Contrary to our prediction, participants who headed soccer balls displayed significantly *higher* beta CMC from pre- to post-test whereas those heading virtual soccer balls in VR showed no change. While this is the first study to show these increases in brain-muscle connectivity, studies have shown evidence of increased connectivity between brain regions in individuals with mTBI and those in the acute phase of concussion (Iraji et al., 2015; Boshra et al., 2020). It is proposed that this hyperconnectivity represents the brain’s natural repair response to injury which then drives compensatory plasticity and modification of brain functional connectivity (Iraji et al., 2015). In essence, after injury, the brain facilitates a search for suitable ways of rerouting information processing to compensate for the reduced cognitive functioning that resulted from injury (Boshra et al., 2020). Although increased CMC has previously been associated with more precise force control (Kristeva et al., 2007; Parr et al., 2023), our data suggests this increased connectivity was not fundamental to task precision (i.e., both groups improved on our force control task). However, there is evidence that CMC increases when individuals are required to exert increased cognitive engagement to maintain isometric force precision in the face of distraction (Safri et al., 2006). Elevated CMC observed in the Heading group may therefore reflect similar increases in cognitive engagement to compensate for processing deficits and/or potentially distracting concussive symptoms of pain and discomfort.

Alternatively, altered CMC following heading may reflect fundamental disruptions to inhibitory motor control. Indeed, a bout of 20 headers has previously been shown to delay the onset of EMG activity following a single pulse of TMS over the primary motor cortex (Di Virgilio et al., 2016), indicating increased corticomotor inhibition and possibly elevated GABA neurotransmitter activity (McDonnell et al., 2006). However, our findings do not indicate increased inhibitory activity *per se*, but potentially a disruption to the spinal modulation of corticospinal loop activity. For example, elevated inhibition of GABA mediated interneurons has been positively associated with the magnitude of spectral EEG beta activity (Porjesz et al., 2002; Baker and Baker, 2003; Matsuya et al., 2017), yet the Heading group displayed no increase in cortical beta activity. By contrast, the magnitude of beta CMC has been negatively associated with the strength of recurrent inhibition generated by spinal Renshaw cells (Matsuya et al., 2017), whereby greater CMC is observed with reduced recurrent inhibition. It is proposed that recurrent inhibition serves to regulate motoneuron excitability and stabilize firing rate by filtering out cortical oscillatory activity at 10 and 20 Hz (Williams and Baker, 2009). Elevated beta CMC following heading, in the absence of elevated spectral EEG beta activity, may therefore reflect a reduced ability to modulate (or filter) the extent to which cortical beta activity becomes propagated to the periphery.

### 4.1. Limitations

There are several limitations that need to be considered when interpreting these data. For example, while the heading impact forces elicited in this study (∼14 *g*) are closely aligned with previous research (Di Virgilio et al., 2016), and the mean reported in professional soccer (∼17 *g*) (The Football Association, 2021b), there is evidence that elite football players might be frequently exposed to head impacts of up to 40 *g* (The Football Association, 2021b). We therefore might expect a similar pattern of impairments but of a greater magnitude at higher ball velocities. We also utilized a ball pressure of 16.2 psi to match recent work showing heading to impair cognitive function (Ashton et al., 2021). Whilst this pressure reflects the upper limit recommended by IFAB, it exceeds the max pressure of 15.2 psi recommended by the FA and may therefore question the generalisability of our findings. However, recent evidence from simulations indicate that the force experienced by the head is more impacted by the speed of the ball rather than its mass or stiffness (Tierney et al., 2021). There was also no follow-up testing to ascertain when these changes in cortical and corticomuscular function dissipated. Based on similar research (Di Virgilio et al., 2016) we would tentatively predict that these cortical and corticomuscular changes will dissipate after 24 h, but this needs exploration in future studies. A limitation of our cognitive assessment was that our experimenter was not blinded when manually timing King-Devick performance, introducing potential bias. Whilst the increased number of performance errors in the Heading group reaffirms that cognitive performance was impaired, future work should attempt to enforce tighter control of these measures. Alternatively, future work could seek to adopt more automated and objective cognitive tasks. Whilst we have shown that cortical activity appears altered following soccer heading, any co-occurring physiological responses to these head impacts remain unclear. For example, there is evidence that sports related concussion can induce acute alterations in cerebral blood flow (Wang et al., 2016; Churchill et al., 2017), a response to head impact that could contribute to the adverse effects of soccer heading. Finally, it has been proposed that female soccer players have an increased susceptibility to concussive events (Sanderson, 2021). While we did not intend to analyze these data by sex, exploratory analyses suggested that sex had no influence on cognitive function, EMG activity or corticomuscular coherence after heading (see text footnote 1). Nevertheless, sex-based differences in the response to repetitive soccer heading warrants further examination in future work.

## 5. Conclusion

In conclusion, we show that a short bout of soccer heading not only impairs cognitive performance, but also induces altered cortical and corticomuscular control of movements. Our findings therefore contribute to a growing body of literature suggesting that soccer heading can induce acute alterations to neurophysiological activity. Whilst we suggest that this altered activity may reflect increased cognitive effort to maintain motor proficiency and/or fundamental disruptions to corticomuscular function, any explanation at this point remains speculative. Further work is therefore required to elucidate the specific nature of these cognitive and neurophysiological adaptations to soccer heading impacts. Future work should also examine how transient these changes in brain activity are and examine potential links between these changes and long-term brain health.

## Data availability statement

The original contributions presented in this study are included in the article/supplementary material, further inquiries can be directed to the corresponding author.

## Ethics statement

The studies involving human participants were reviewed and approved by the Manchester Metropolitan University Ethics Committee. The patients/participants provided their written informed consent to participate in this study.

## Author contributions

JP and GW: data curation, formal analysis, and writing—original draft. JP, LU, and GW: project administration, supervision, and validation. JP and BM: software. JP: visualization. All authors: conceptualization, methodology, writing—review and editing, read, and approve manuscript.

## Footnotes

1
https://osf.io/8947h/?view_only=9a9899e563634d8bb09a2700550d3012

2
http://seniam.org/sensor_location.htm
